# Cognitive Diagnostic Models for Random Guessing Behaviors

**DOI:** 10.3389/fpsyg.2020.570365

**Published:** 2020-09-25

**Authors:** Chia-Ling Hsu, Kuan-Yu Jin, Ming Ming Chiu

**Affiliations:** ^1^Assessment Research Centre, The Education University of Hong Kong, Tai Po, Hong Kong; ^2^Hong Kong Examinations and Assessment Authority, Wan Chai, Hong Kong

**Keywords:** response time, rapid guessing, G-DINA model, DINA model, DINO model

## Abstract

Many test-takers do not carefully answer every test question; instead they sometimes quickly answer without thoughtful consideration (*rapid guessing*, RG). Researchers have not modeled RG when assessing student learning with *cognitive diagnostic models* (CDMs) to personalize feedback on a set of fine-grained skills (or attributes). Therefore, this study proposes to enhance cognitive diagnosis by modeling RG via an advanced CDM with item response and response time. This study tests the parameter recovery of this new CDM with a series of simulations via Markov chain Monte Carlo methods in JAGS. Also, this study tests the degree to which the standard and proposed CDMs fit the student response data for the Programme for International Student Assessment (PISA) 2015 computer-based mathematics test. This new CDM outperformed the simpler CDM that ignored RG; the new CDM showed less bias and greater precision for both item and person estimates, and greater classification accuracy of test results. Meanwhile, the empirical study showed different levels of student RG across test items and confirmed the findings in the simulations.

## Introduction

Cognitive diagnostic models (CDMs) assess whether test-takers have the skills needed to answer test questions (*attributes*), so that their test results can give them diagnostic feedback on their strengths and weaknesses in these attributes (Rupp et al., 2010). Specifically, a CDM analysis determines whether a person shows mastery (vs. non-mastery) of a set of fine-grained attributes (*latent class*). Teachers, clinicians and other users of test scores can use such specific information on each student or client to adapt and improve their instructions/interventions more effectively, compared to a simple, summative score.

However, some test-taking behaviors can distort current CDM results and thereby jeopardize the validity of their assessments. Recently, researchers have proposed different approaches to account for test-taking behaviors when assessing test-taker performance and item characteristics. In this study, we focus on two frequently-observed test-taking behaviors during actual tests: solution attempt and *rapid guessing* (RG; Wise and Kong, 2005). In a solution attempt, test-takers carefully try to find answers to test questions. By contrast, RG refers to test-takers quickly answering test questions without thoughtful consideration (e.g., Wise and DeMars, 2006). For instance, Meyer (2010) integrated a two-class mixture Rasch model (Rost, 1990) to classify a test-taker as either making a solution attempt or RG, but not allowing different behaviors by the same person during a test. To address this limitation, Wang and Xu (2015) proposed a model with a latent indicator to allow each test-taker to engage in either a solution attempt or RG on each item. Furthermore, the indicator can depend on either a test-taker’s RG propensity or on an item-level feature (Wang et al., 2018). As RGs are typically much shorter than solution attempts, CDMs can use a test-taker’s reaction time (RT) to each test question to properly model RGs and distinguish them from solution attempts (but not necessarily pre-knowledge answers, e.g., Wang et al., 2018). As no published study has proposed and tested a CDM that models RG, we do so in this study.

This study proposes a new framework of CDMs to recognize different test-taking behaviors by using RT and item responses simultaneously. This new class of CDMs: (1) models two test-taking behaviors (RG vs. solution attempt) for each item-person concurrence, (2) allows multiple switch points between RG and solution attempts among the items for each test-taker, (3) thereby yields person and item estimates with greater accuracy, and (4) generalizes to available CDMs, RT functions and other kinds of dissimilar behaviors.

The generalized DINA model (G-DINA, de la Torre, 2011) conceptualizes and shows the utility of this framework. Specifically, the two special cases of the G-DINA model, the deterministic input, noisy “and” gate (DINA) model (Junker and Sijtsma, 2001) and its counterpart, the deterministic input, noisy “or” gate (DINO) model (Templin and Henson, 2006) are simple to compute, estimate, and interpret, so they serve as illustrations. Nevertheless, researchers can extend this approach to other CDMs, especially G-DINA-liked formulation CDMs, such as the general diagnostic model (GDM; von Davier, 2005) and the linear logistic model (Maris, 1999).

After we present the functions for describing RT and item response, we specify the new model. Next, our simulation study illustrates the new model’s performance, followed by its application to real data. Lastly, we discuss the implications of this study for identifying test-taking behaviors and improving the estimation accuracy of both person and item parameters.

## A New CDM Framework

The new model requires distinct functions to separately specify two fundamentals for an item, RT and item response, while two main facets, person and item, affect the observed RT and item response. This section describes the adopted RT and item response functions, before specifying the new model.

### The Lognormal RT Model

As cognitive test data typically resemble a lognormal distribution more closely than a normal distribution, we use a lognormal function to characterize RT (van der Linden, 2006, 2007). Let *RT*_*ij*_ be the observed RT of person *i* (*i* = 1, 2, …, *I*) to item *j* (*j* = 1, 2, …, *J*). In the lognormal function, the two parameters of *person speed* and *time intensity*, respectively, represent the two facts, person and item, as follows,

(1) $$log\left(RT_{ij}\right)∼N\left(\beta_{j}-\tau_{i},1/\kappa_{j}^{2}\right)$$

where τ_*i*_ indicates the average speed of test-taker *i* on a test (person speed); β_*j*_ indicates the mean time that the population needs to resolve item *j* (time intensity); and $\kappa_{j}^{2}$ indicates the dispersion of the logarithmized RT distribution (time discrimination parameter) of item *j*.

### The G-DINA Model

The G-DINA model loosens some restrictions of the DINA model and its saturated form is equivalent to other general CDMs via link functions (de la Torre, 2011). Hence, the G-DINA model can (a) present different CDMs with similar formulations via various constraints and (b) substantially reduce the number of latent classes for an item – especially for models with more than five attributes. The original G-DINA model with identity link can be expressed as

$$P\left(\alpha_{ij}^{*}\right)=\delta_{j0}+\sum_{k=1}^{K_{j}^{*}}\delta_{jk}\alpha_{ik}+\sum_{k^{\prime }=k+1}^{K_{j}^{*}}$$

(2) $$\sum_{k=1}^{K_{j}^{*}-1}\delta_{jkk^{\prime }}\alpha_{ik}\alpha_{ik^{\prime }}\cdots +\delta_{j12\cdots K_{j}^{*}}\prod_{k=1}^{K_{j}^{*}}\alpha_{ik}$$

For test-taker *i*, the reduced attribute vector $\alpha_{ij}^{*}$ has the required attributes for item *j*. The intercept for item *j*, δ_*j*0_ represents the probability of a correct response without the required attributes (*baseline probability*). The main effect δ_*jk*_ reflects the extent to which mastery of a single attribute α_*k*_changes the probability of a correct response. The interaction effect δ_*jkk*′_ indicates the extent to which mastery of both attributes α_*k*_ and $\alpha_{k^{\prime }}$ changes the probability of a correct response. The interaction effect $\delta_{j12\cdots K_{j}^{*}}$ reflects the extent to which mastery of all the required attributes α_1_, α_2_, ⋯, and $\alpha_{K_{j}^{*}}$ changes the probability of a correct response.

Like most CDMs, the G-DINA model requires a *J*×*K* Q-matrix (Tatsuoka, 1983), in which *K* knowledge attributes are required to correctly answer *J* items. $K_{j}^{*}=\sum_{k=1}^{K}q_{jk}$ is the number of required attributes for item *j*, where *q*_*jk*_ = 1 if the correct response to item *j* requires attribute *k*; and 0 otherwise. As the number of required attributes for item *j* is smaller than that of the all attribute vectors ($K_{j}^{*}<K$), the G-DINA model can reduce the number of required latent classes ($2^{K_{j}^{*}}$ < 2^*K*^) for an item. To illustrate the G-DINA-like formulations, we use two common cases: the DINA and DINO.

#### The DINA Model

In the non-compensatory DINA, individuals are classified into one of two latent classes for an item: (a) the attribute vectors have all of an item’s required attributes (*mastery*) or (b) the attribute vectors are missing at least one of the item’s required attributes (*non-mastery*). The two latent classes’ corresponding probabilities for a correct response entail that (a) mastery individuals do not slip, or (b) non-mastery individuals guess correctly (Junker and Sijtsma, 2001). Thus, the DINA model can be re-formed by setting to zero, all G-DINA model parameters except δ_*j*0_ and $\delta_{j12\cdots K_{j}^{*}}$

(3) $$P\left(\alpha_{ij}^{*}\right)=\delta_{j0}+\delta_{j12\cdots K_{j}^{*}}\prod_{k=1}^{K_{j}^{*}}\alpha_{ik}$$

In Eq. 3, δ_*j*0_ = *g*_*j*_ is the probability of a correct response to item *j* for a non-mastery test-taker *i*, where *g*_*j*_ is the guessing parameter for item *j*; $\delta_{j0}+\delta_{j12\cdots K_{j}^{*}}=1-s_{j}$ is the probability of a correct response to item *j* for a mastery test-taker *i*, where *s*_*j*_ is the slipping parameter for item *j*. In the DINA, a mastery test-taker with all the required attributes ($K_{j}^{*}$) for item *j* generally answers it correctly and other test-takers generally answer it incorrectly. Like the DINA, Eq. 3 shows that except for the attribute vector $\alpha_{j}^{*}=1_{K_{j}^{*}}$ (in which $1_{K_{j}^{*}}$ is a vector of ones with length $K_{j}^{*}$), other latent classes ($2^{K_{j}^{*}}-1$) have the same probability of correctly answering item *j*. As shown in Eq. 3, this probability increases only after mastering all the required attributes. Under the DINA model assumption (Junker and Sijtsma, 2001), the G-DINA has two parameters per item (see Eq. 3).

#### The DINO Model

Unlike the non-compensatory DINA, the compensatory DINO only entails at least one of the required attributes to answer an item, so the parameters in G-DINA are set to

(4) $$\delta_{jk}=\left(-1\right)\delta_{jk^{\prime }k^{{}^{\prime \prime }}}=\cdots ={\left(-1\right)}^{K_{j}^{*}+1}\delta_{j1,2,\cdots ,K_{j}^{*}}$$

where $k=1,\cdots ,K_{j}^{*}$, $k^{\prime }=1,2,\cdots K_{j}^{*}-1$, and $k^{{}^{\prime \prime }}>k^{\prime },\cdots ,K_{j}^{*}$. The orders of the interactions vary the alternating sign, and the quantities of the main effects and interactions have the same value.

(5) $$P\left(\alpha_{ij}^{*}\right)=\delta_{j0}+\delta_{jk}\alpha_{ik}.$$

For a test-taker *i* with at least one of the required attributes, the probability of answering item *j* without slipping ($s_{j}^{\prime }$) is $\delta_{j0}+\delta_{jk}=1-s_{j}^{\prime }$. Likewise, for a test-taker *i* with none of the required attributes, the probability of correctly answering item *j* is the guessing parameter, $\delta_{j0}=g_{j}^{\prime }$. Unlike the DINA, all latent classes except for the attribute vector $\alpha_{j}^{*}=0_{K_{j}^{*}}$ (a vector of zeros and of length $K_{j}^{*}$) have the same probability of correctly answering item *j*. Like the DINA, the DINO only needs two parameters for an item (Eq. 5, Templin and Henson, 2006).

To use both information of RT and item response, two functions must be specified. Hence, RT-GDINA, RT-DINA and RT-DINO jointly model RT and item response with the lognormal distribution (Eq. 1) and G-DINA, DINA and DINO, respectively.

### New Class of CDMs

We introduce a new class of G-DINA to account for varying test-taking behaviors. RT (*RT*_*ij*_) and item response (*Y*_*ij*_) are modeled individually. As test-takers can switch between RG and solution behaviors, like Wang and Xu (2015), a latent indicator (ξ) is employed, where if test-taker *i* tries to solve item *j*, ξ_*ij*_ = 1 (0 otherwise; RG is specified in this study). Incorporating this latent indicator into the lognormal RT model extends Eqs 1–6

(6) $$\left\{ \begin{array}{cc} log\left(RT_{ij}\right)∼N\left(\beta_{j}-\tau_{i},1/\kappa_{j}^{2}\right), & \mathrm{if}\xi_{ij}=1;\\ log\left(RT_{ij}\right)∼N\left(\beta_{0},1/\kappa_{0}^{2}\right), & \text{if}\xi_{ij}=0. \end{array}\right.$$

indicates that the logarithmized RT is normally distributed as Eq. 1 if test-taker *i* solves item *j* from solution attempt (ξ_*ij*_ = 1), and it is normally distributed with mean time intensity β_0_ and time discrimination $\kappa_{0}^{2}$ if test-taker *i* responds to item *j* with a RG (ξ_*ij*_ = 0). For a RG on item *j* by test-taker *i*, RT is constant.

Likewise, adding ξ_*ij*_ to the G-DINA yields

$$P\left(\alpha_{ij}^{*}\right)=\xi_{ij}(\delta_{j0}+\sum_{k=1}^{K_{j}^{*}}\delta_{jk}\alpha_{ik}+\sum_{k^{\prime }=k+1}^{K_{j}^{*}}$$

(7) $$\sum_{k=1}^{K_{j}^{*}-1}\delta_{jkk^{\prime }}\alpha_{ik}\alpha_{ik^{\prime }}\cdots +\delta_{j12\cdots K_{j}^{*}}\prod_{k=1}^{K_{j}^{*}}\alpha_{ik})+(1-\xi_{ij})\delta {}_{j}{}^{*}$$

The G-DINA model is the underlying model for a solution attempt on item *j* by test-taker *i* (ξ_*ij*_ = 1). We assume that a RG on item *j* by test-taker *i* (ξ_*ij*_ = 0) yields $\delta_{j}^{*}$. For simplicity, like Wise and DeMars (2006), we assume that test-taker *i* has the same probability of correctly answering item *j* both by RG and by guessing with none of the required attributes ($\delta_{j}^{*}=\delta_{j0}$), that is, guessing randomly for all options. Hence, Eq. 7 can be re-written as

$$P\left(\alpha_{ij}^{*}\right)=\delta_{j0}+\xi_{ij}(\sum_{k=1}^{K_{j}^{*}}\delta_{jk}\alpha_{ik}+\sum_{k^{\prime }=k+1}^{K_{j}^{*}}$$

(8) $$\sum_{k=1}^{K_{j}^{*}-1}\delta_{jkk^{\prime }}\alpha_{ik}\alpha_{ik^{\prime }}\cdots +\delta_{j12\cdots K_{j}^{*}}\prod_{k=1}^{K_{j}^{*}}\alpha_{ik})$$

The latent indicator ξ_*ij*_ in Eqs 6–8 is a binary result of test-taker *i* on item *j*’s behavior (solution attempt vs. RG). It can be modeled by a Bernoulli distribution with π_*j*_, the marginal probability of the solution attempt. Using the DINA and DINO to identify RGs and solution attempts, Eqs 3 and 5 are re-written, respectively, as Eqs 8 and 9.

(9) $$P\left(\alpha_{ij}^{*}\right)=\delta_{j0}+\xi_{ij}\delta_{j12\cdots K_{j}^{*}}\prod_{k=1}^{K_{j}^{*}}\alpha_{ik}$$

(10) $$P\left(\alpha_{ij}^{*}\right)=\delta_{j0}+\xi_{ij}\delta_{jk}\alpha_{ik}$$

If test-taker *i* tries to solve item *j* (ξ_*ij*_ = 1), Eqs 6, 8–10 reduce, respectively, to Eqs 1–3, and 5. Thus, the lognormal, G-DINA, DINA, and DINO are special cases of our proposed new CDM framework. Likewise, jointly modeling RT and item response with a latent indicator via Eqs 6 and 8–10 are, respectively, represented as RT-GDINA-RG, RT-DINA-RG and RT-DINO-RG.

To illustrate this approach, we combine the lognormal RT distribution and the easy-to-understand DINA and DINO models, with the latent indicator ξ (RT-DINA-RG, RT-DINO-RG) and without it (RT-DINA, RT-DINO). We estimate their parameters via the Bayesian method with the Markov chain Monte Carlo (MCMC) algorithm in the freeware JAGS (Plummer, 2017). For the JAGS code and the priors for the estimated parameters of the RT-DINA-RG and RT-DINO-RG models (see Appendix).

## Simulation Study 1: Parameter Recovery of RT-DINA-RG

### Design

In simulation study 1, we evaluated the parameter recovery of the RT-DINA-RG for a test of 30 dichotomous items measuring five non-compensatory attributes. See the artificial Q-matrix in Table 1. The guessing (*g*_*j*_) and slipping (*s*_*j*_) parameters were randomly generated, respectively, from the uniform distributions of *U*(0.05, 0.3) and *U*(0.05, 0.2), which reflect a high quality test. This data-generating procedure for the 30 simulated items yielded item discrimination indices (IDI) that ranged from 0.51 to 0.88, indicating a test with high measurement quality (Lee et al., 2012).

**TABLE 1:** Specified Q-matrix and item parameters in simulation 1.

Item	*q*_1_	*q*_2_	*q*_3_	*q*_4_	*q*_5_	π*_*j*_*
1	1	0	0	0	0	0.9
2	0	1	0	0	0	0.9
3	0	0	1	0	0	0.9
4	0	0	0	1	0	0.9
5	0	0	0	0	1	0.9
6	1	0	0	0	0	0.8
7	0	1	0	0	0	0.8
8	0	0	1	0	0	0.8
9	0	0	0	1	0	0.8
10	0	0	0	0	1	0.8
11	1	1	0	0	0	0.9
12	1	0	1	0	0	0.9
13	1	0	0	1	0	0.9
14	1	0	0	0	1	0.9
15	0	1	1	0	0	0.9
16	0	1	0	1	0	0.8
17	0	1	0	0	1	0.8
18	0	0	1	1	0	0.8
19	0	0	1	0	1	0.8
20	0	0	0	1	1	0.8
21	1	1	1	0	0	0.9
22	1	1	0	1	0	0.9
23	1	1	0	0	1	0.9
24	1	0	1	1	0	0.9
25	1	0	1	0	1	0.9
26	1	0	0	1	1	0.8
27	0	1	1	1	0	0.8
28	0	1	1	0	1	0.8
29	0	1	0	1	1	0.8
30	0	0	1	1	1	0.8

We manipulated two conditions. In the RG condition, the marginal probability of RG (1 − π_*j*_) was set for items at two levels: 0.1 and 0.2 (Wang et al., 2018). To describe the dynamic latent indicator of person *i* on item *j* in the RG condition, the ξ-parameter was generated from a Bernoulli distribution with probability either of 0.8 or 0.9. In the RT-DINA-RG, mean item time intensity (β_0_) and item discrimination (κ_0_) were (a) set, respectively, at 2 and 1.6 for rapid guessers (ξ_*ij*_ = 0) and (b) generated, respectively, from *U*(2, 4) and *U*(0.15, 2) for normal test-takers (ξ_*ij*_ =  1). In non-RG condition, RG never occurs, and the RT-DINA served as the data-generating model, yielding parameters similar to the RT-DINA-RG. Mean item time intensity and item discrimination can be generated to accommodate various test situations (e.g., Man et al., 2018), but they do not affect the use of the proposed model. Therefore, we leave this interesting topic for further study.

We simulated 1,000 test-takers across conditions, and each test-taker had generated five latent attributes with positive correlations, following Henson and Douglas (2005) procedure. Specifically, we randomly generated 1,000 vectors with five values, α_*i*_ = (α_*i*1_, α_*i*2_, α_*i*3_, α_*i*4_, α_*i*5_)′, *i* = 1, 2, …, 1,000, from a multivariate normal distribution with no interaction, *MVN*(**0.5**, Σ) with Σ diagonal elements of 1.0 and others of 0.5. A cut-off value of 0.253 (z_0__.6_) indicated mastery of the attribute (if α_*ik*_ > 0.253, α_*ik*_ = 1; otherwise, α_*ik*_ = 0), yielding ∼60% mean mastery of each attribute, which generally ranged from easy to moderate. The person speed parameter (τ_*i*_) was generated from *N*(0, 0.3^2^). Each condition was replicated 100 times from an R script.

Both the RT-DINA and RT-DINA-RG were fit to these data to test three hypotheses: (1) with some RG, the RT-DINA-RG efficiently recovers item and person estimates; (2) ignoring RG via the RT-DINA yields biased item parameter estimates, less accurate classification of attribute mastery, and less reliable person speed estimates; and (3) with no RG, the RT-DINA-RG performs as well as the RT-DINA. To evaluate the recovery of item parameters, the bias and root mean squared error (RMSE) were computed as dependent variables:

(11) $$\text{Bias}\left(\hat{\nu }\right)=\sum_{r=1}^{100}\left({\hat{\nu }}_{r}-\nu \right)/100$$

(12) $$\text{RMSE}\left(\hat{\nu }\right)=\sqrt{\sum_{r=1}^{100}{\left({\hat{\nu }}_{r}-\nu \right)}^{2}/100}$$

where ν and ${\hat{\nu }}_{r}$ indicate respectively, true and estimated values in the *r*-th replication of an item parameter. We examined test-takers’ true and estimated latent classes to evaluate the classification accuracy of each attribute. The reliability of the person speed parameter was computed as:

(13) $$\text{Reliability}\left(\hat{\tau }\right)=\text{Correlation}{\left(\hat{\tau },\tau \right)}^{2}$$

### Results

**FIGURE 1:**
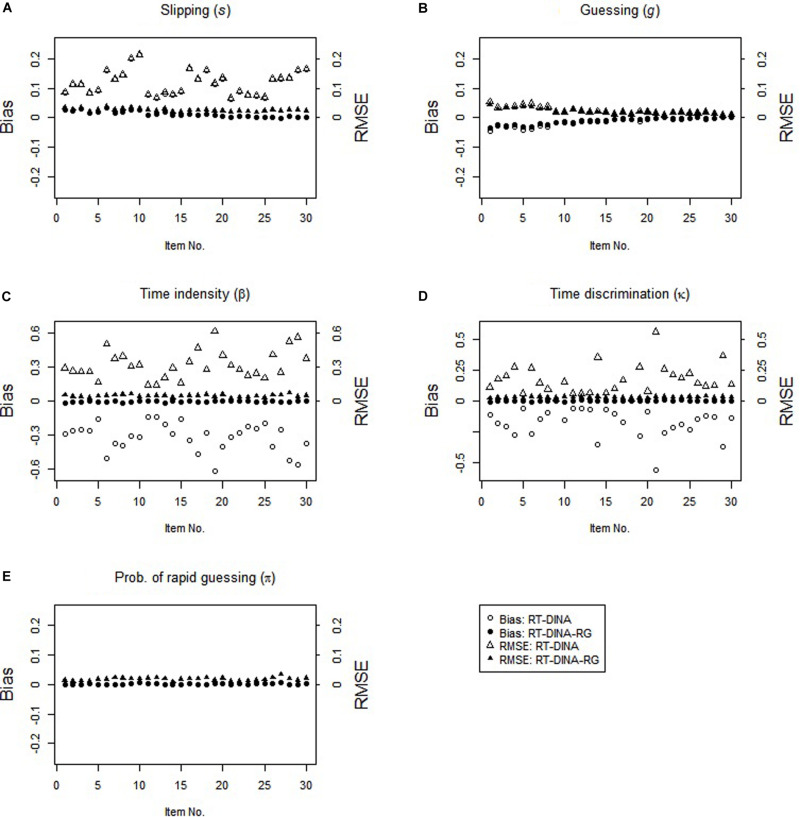
Parameter recovery for the RT-DINA and RT-DINA-RG under the RG condition in simulation 1.

In the RG condition, the RT-DINA-RG generally yielded unbiased parameter estimates, whereas the RT-DINA overestimated the slipping parameters and underestimated the item intensity, guessing, and time discrimination parameters (see Figure 1). Greater RG increased the severities of slipping overestimation and time intensity underestimation. For test-takers without the required attributes, RG did not influence the success rate, so ignoring RG did not substantially influence estimation of the guessing parameters. Across five attributes and 100 replications, mean classification accuracy was higher for the RT-DINA-RG than the RT-DINA (0.936 > 0.924), suggesting that ignoring RG reduces the accuracy of attribute classification. Also, the RT-DINA-RG outperformed the RT-DINA on reliability of the person speed parameter (*M*: 0.66 > 0.57).

**FIGURE 2:**
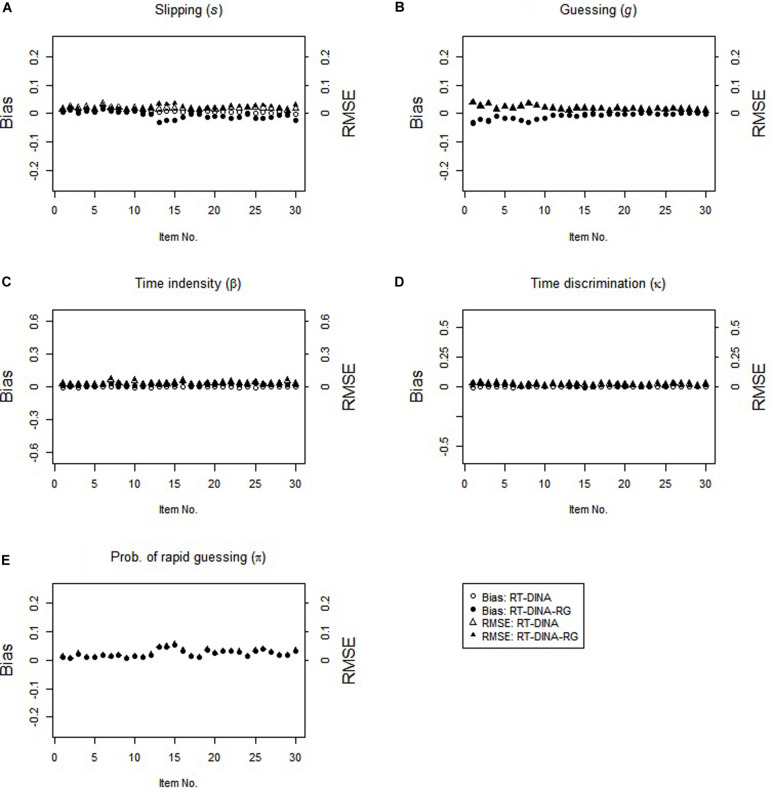
Parameter recovery for the RT-DINA and RT-DINA-RG under the non-RG condition in simulation 1.

In the non-RG condition, both RT-DINA and RT-DINA-RG recovered the parameters well (see Figure 2). The bias and RMSE for the π-parameter in the RT-DINA-RG were nearly zero. Also, both models yielded practically identical classification accuracy (*M* = 96.6%) and reliability of person speed parameter (*M* = 0.76) across 100 replications. Hence, overfitting the RT-DINA-RG to data without RG showed no significant harm. In brief, the simulation results supported our three hypotheses.

## Simulation Study 2: Parameter Recovery of RT-DINO-RG

### Design

Study 2 simulated compensatory attributes and analyzed parameter recovery by the RT-DINO and RT-DINO-RG. The item responses and RTs were generated for (a) the RG condition with the RT-DINO-RG and (b) the non-RG condition with the RT-DINO. The parameters, data generation and evaluation criteria were the same as those in simulation study 1. Paralleling study 1, we test three hypotheses: (1) with some RG, the RT-DINO-RG efficiently recovers item and person estimates; (2) ignoring RG via the RT-DINO yields biased item parameter estimates and less accurate classification of attribute mastery; and (3) with no RG, the RT-DINO-RG performs as well as the RT-DINO.

### Results

**FIGURE 3:**
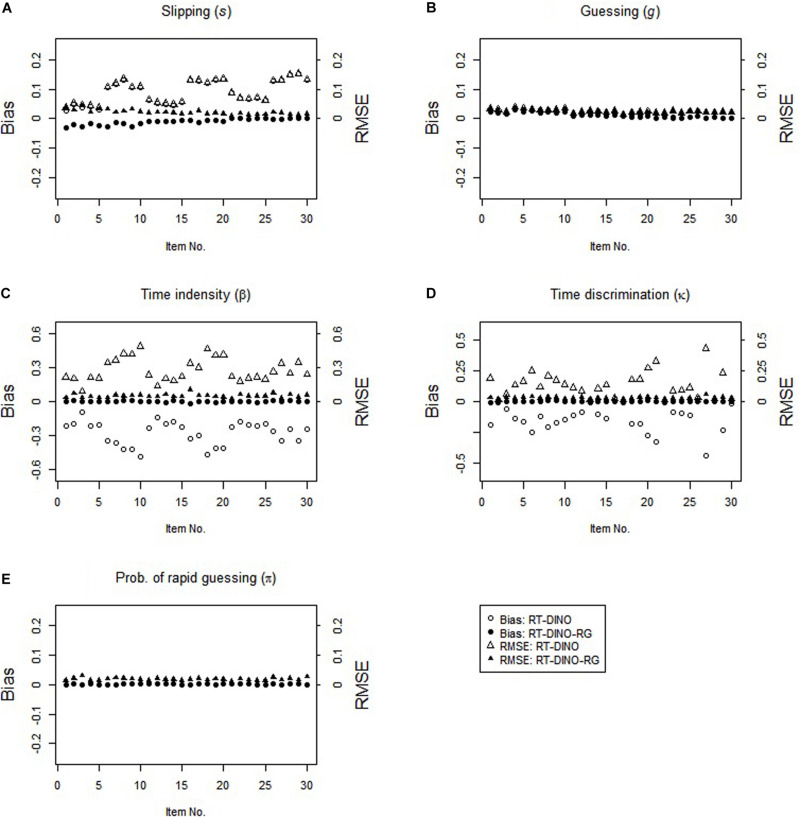
Parameter recovery for the RT-DINO and RT-DINO-RG under the RG condition in simulation 2.

The study 2 results resemble the study 1 results (see Figure 3). In the RG condition, the RT-DINO-RG recovered the parameters well, whereas the RT-DINO overestimated the slipping parameters and underestimated the item intensity, guessing, and time discrimination parameters. Greater RG increased the severities of slipping overestimation and time intensity underestimation. The RT-DINO-RG outperformed the RT-DINO on both mean classification accuracy (0.946 > 0.922) across five attributes and reliability of the person speed parameter (*M*: 0.64 > 0.57).

**FIGURE 4:**
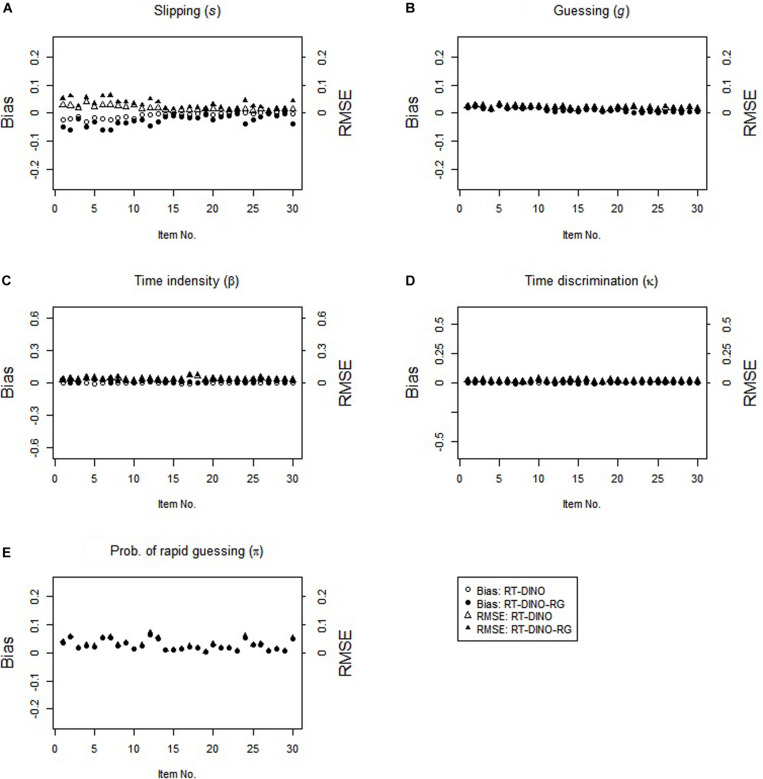
Parameter recovery for the RT-DINO and RT-DINO-RG under the non-RG condition in simulation 2.

In the non-RG condition, both RT-DINO and RT-DINO-RG recovered the item parameters well (see Figure 4). The bias and RMSE for π-parameter in the RT-DINO-RG model were very small. Also, both models had practically identical classification accuracy (*M* = 98.4%) and reliability of person speed parameter (*M* = 0.71) across replications. In sum, these simulation results supported our three hypotheses.

## Real Data Analysis

To illustrate a RT-GDINA-RG application, we analyzed a PISA 2015 mathematics test with 22 questions. After screening out students with missing responses, we analyzed 5,158 students’ responses. The PISA 2015 mathematics assessment framework (OECD, 2017a, b) and the released computer-based mathematics items’ log-file databases covered eight attributes: change and relationships (α_1_), quantity (α_2_), space and shape (α_3_), uncertainty (α_4_), occupational (α_5_), societal (α_6_), scientific (α_7_), and personal (α_8_). The Q-matrix for the mathematics test shows two cognitive attributes for each item (see Table 2). We fit the four CDM models (RT-DINA, RT-DINO, RT-DINA-RG, RT-DINO-RG) to these data. Superior models have lower deviance information criteria (DIC; Spiegelhalter et al., 2002).

**TABLE 2:** Specified Q-matrix for the real data.

Item	Label	*q*_1_	*q*_2_	*q*_3_	*q*_4_	*q*_5_	*q*_6_	*q*_7_	*q*_8_
1	CM033Q01S	0	0	1	0	0	0	0	1
2	CM474Q01S	0	1	0	0	0	0	0	1
3	DM155Q02C	1	0	0	0	0	0	1	0
4	CM155Q01S	1	0	0	0	0	0	1	0
5	DM155Q03C	1	0	0	0	0	0	1	0
6	CM155Q04S	1	0	0	0	0	0	1	0
7	CM411Q01S	0	1	0	0	0	1	0	0
8	CM411Q02S	0	0	0	1	0	1	0	0
9	CM803Q01S	0	0	0	1	1	0	0	0
10	CM442Q02S	0	1	0	0	0	1	0	0
11	DM462Q01C	0	0	1	0	0	0	1	0
12	CM034Q01S	0	0	1	0	1	0	0	0
13	CM305Q01S	0	0	1	0	0	1	0	0
14	CM496Q01S	0	1	0	0	0	1	0	0
15	CM496Q02S	0	1	0	0	0	1	0	0
16	CM423Q01S	0	0	0	1	0	0	0	1
17	DM406Q01C	0	0	1	0	0	1	0	0
18	DM406Q02C	0	0	1	0	0	1	0	0
19	CM603Q01S	0	1	0	0	0	0	1	0
20	CM571Q01S	1	0	0	0	0	0	1	0
21	CM564Q01S	0	1	0	0	0	1	0	0
22	CM564Q02S	0	0	0	1	0	1	0	0

The results indicate both compensatory attributes and RG. DICs showed that the compensatory models outperformed the non-compensatory ones (RT-DINO < RT-DINA: 1,351,697 < 1,433,173; and RT-DINO-RG < RT-DINA-RG: 1,327,068 < 1,360,978) suggesting that the eight attributes’ relationships were more compensatory than non-compensatory. Also, the RG models outperformed the simpler models (RT-DINO-RG < RT-DINO: 1,327,068 < 1,351,697; and RT-DINA-RG < RT-DINA: 1,360,978 < 1,433,173), showing substantial RG. As the data indicated both compensatory attributes and RG, the RT-DINO-RG showed the best fit. Hence, we examine the RT-DINO and RT-DINO-RG results in greater detail.

**FIGURE 5:**
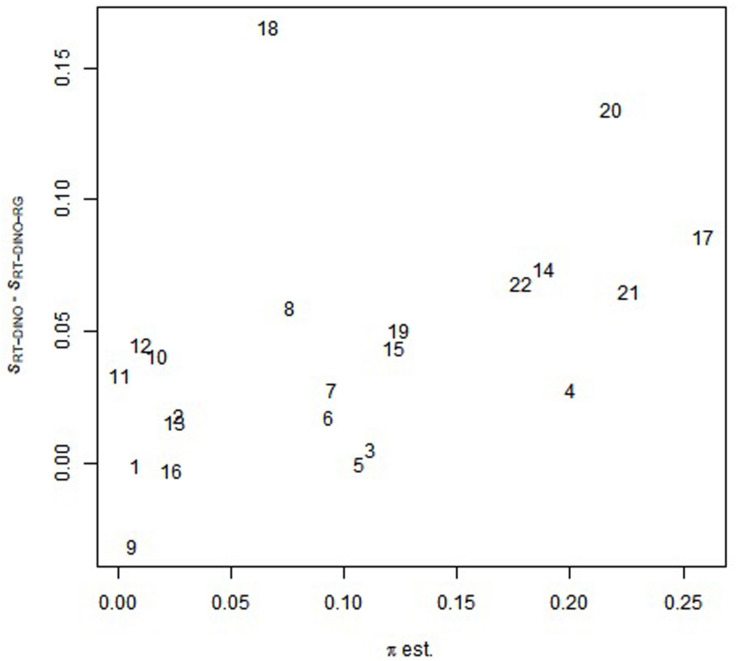
Relationship between π-parameter in the RT-DINO-RG and the difference in the slipping parameters between the RT-DINO the RT-DINO-RG models. Numbers are item identifiers; πEst is RG estimates; and *S*_RT-DINO_ – *S*_RT-DINO-RG_ is the difference in the slipping parameters of RT-DINO and RT-DINO-RG.

Like study 2, the RT-DINO estimated higher slipping parameters and lower guessing parameters, compared to the RT-DINO-RG (slipping: *M*_RT-DINO_ > *M*_RT-DINO-RG_: 0.27 > 0.22; guessing: *M*_RT-DINO_ < *M*_RT-DINO-RG_: 0.28 < 0.30). Also, the mean discrimination power of RT-DINO-RG exceeded that of RT-DINO (IDI*_*M*_*_(RT-DINO-RG)_ > IDI*_*M*_*_(RT-DINO)_: 0.47 > 0.45). Ranging from 0.74 to 0.99, RT-DINO-RG’s RG estimates (π) moderately correlated (*r* = 0.49) with the difference in the slipping parameters of RT-DINO and RT-DINO-RG (see Figure 5), supporting the simulation study 2 finding of overestimated slipping parameters when ignoring RGs. Also, the πs of items 1–11 were generally lower than those of items 12–22. If these items appeared on the test in this sequence (item position information was not publicly available), these π results suggest that test-taker accuracy depended on their completion speed (*speededness*).

**FIGURE 6:**
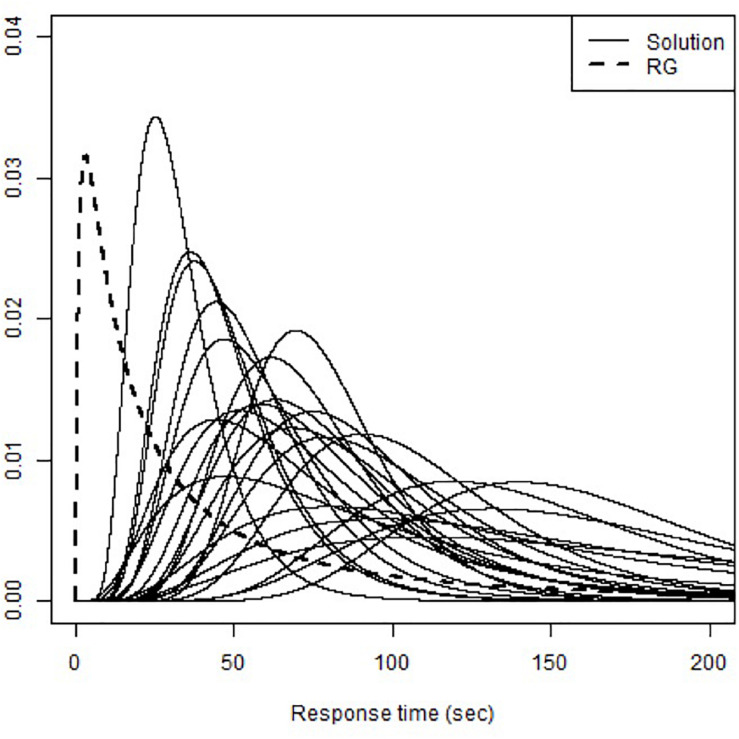
Probability density function of RT for the RT-DINO-RG.

The RT-DINO-RG also uses response time to recognizes RGs and solution attempts, showing estimated mean time intensity (β_0_) of 3.21 and time discrimination (κ_0_) of 0.70. The various probability density functions of response time for RGs and solution attempts in the RT-DINO-RG (see Figure 6) suggest that students used varied answering strategies to spend more time on some items and less time on others (including RGs). The RT-DINO and RT-DINO-RG did not consistently classify mastery of the eight attributes [Cohen’s κ ranged from 0.48 (quantity) to 0.98 (occupational), see Table 3]. Notably, few students had knowledge of the third attribute (space and shape). The simulation studies suggest that the RT-DINO-RG classifications are more reliable than the RT-DINO ones.

**TABLE 3:** Mastery of attributes for the RT-DINO and RT-DINO-RG.

	Attributes							
	α_*1*_	α_*2*_	α_*3*_	α_*4*_	α_*5*_	α_*6*_	α_*7*_	α_*8*_
RT-DINO	0.090	0.381	0.057	0.133	0.173	0.235	0.366	0.349
RT-DINO-RG	0.234	0.359	0.036	0.151	0.168	0.166	0.254	0.361
Cohen’s *k*	0.483	0.861	0.644	0.899	0.975	0.762	0.721	0.885

## Discussion

CDMs assess whether test-takers have the needed skills (*attributes*) to answer each test question and give suitable diagnostic feedback, but they have not adequately modeled RG vs. solution attempts with reaction times. Hence, this study developed a new class of CDMs based on the G-DINA model (de la Torre, 2011), namely RT-GDINA-RG, with a latent indicator to jointly utilize both item responses and RTs to model RG and solution attempts to enhance cognitive diagnosis. We propose two models based on the DINA and DINO models, namely RT-DINA-RG and RT-DINO-RG.

The RT-DINA-RG and RT-DINO-RG were evaluated via (a) simulation studies with Markov chain Monte Carlo methods in JAGS and (b) real data analysis by analyzing the PISA 2015 computer-based mathematics test. Complementing Wang and Xu (2015) person-level manipulation of RG, this study manipulated RG at the item level (Wang et al., 2018). The simulation results and real data analysis showed that the RT-DINA-RG and RT-DINO-RG recovered parameters well and assessed test-takers’ diagnostic results more accurately. In contrast, ignoring RGs by fitting simpler models yielded biased parameters, less reliable person speed parameter, and less classification accuracy of test results.

Hence, this study extends research showing how analyses of RT improves cognitive assessments of test-takers (e.g., van der Linden, 2008; Lee and Chen, 2011; Wang and Xu, 2015). When test-takers rapidly guess, the RT-GDINA-RG yields greater accuracies in person parameters, item parameters, and cognitive results. Therefore, researchers or users should use the RT-GDINA-RG to depict a data if RGs might occur. The choice of RT-GDINA-RG model (i.e., RT-DINA-RG or RT-DINO-RG) depends on the nature of the test items. If a test item’s needed underlying constructs can compensate for one another, then RT-DINO-RG is suitable. If the underlying constructs cannot compensate for one another, then RT-DINA-RG is suitable.

Moreover, the person and item parameters of the RT-GDINA-RG were assumed to be, respectively, independent in this study. As attributes and item parameters of a CDM are often related in practice, we capture the relations between them with correlational structures (e.g., van der Linden, 2007). Note that the commonly-used multivariate normal distribution to specify the relations among person parameters is not feasible for the discrete feature of attributes in CDMs. Following Zhan et al. (2018), one can address this problem by using a higher-order latent trait to link the correlated attributes (de la Torre and Douglas, 2004), and then assuming that the person parameters (i.e., the higher-order latent trait and person speed) follow a bivariate normal distribution.

In addition, this study assumes the same probability of correctly answering an item by a RG as by guessing with none of the required attributes for the sake of simplicity. Such a naïve assumption can be further explored as in Wang and Xu (2015). Further, the RT-GDINA-RG distinguishes between solution attempt and RG for cognitive diagnosis via a latent indicator. In addition to RG, RT-GDINA-RG can be easily extended to adapt diverse test-taking behaviors and various tests’ requirements. For example, we can extend CDMs to include other test-taking behaviors such as *prior knowledge/pre-knowledge* (Wang et al., 2018; Man et al., 2019) or *nonresponses* (Ulitzsch et al., 2019) if and only if the probabilities of a correct response from different latent indicators (or classes) can be clearly defined. In a high-stakes test, individuals often use pre-knowledge to correctly answer items with extremely short RT (unlike solution attempts with relatively long RT and unlike RGs with often wrong answers and short RT). Furthermore, we can adapt the functions for depicting RT and item response to the testing contexts, such as linear transformation (Wang et al., 2013), a gamma distribution to depict RT for mental rotation items (Maris, 1993), etc. (De Boeck and Jeon, 2019). Also, the item response function can be replaced by other CDMs, such as the GDM (von Davier, 2005) or the linear logistic model (Maris, 1999). Future studies can investigate these approaches.

In addition, ignoring RGs can harm the development and application of cognitive assessments (for both high- and low-stakes tests), distort test results, or invalidate inferences. For example, greater precision of test parameters via the RT-GDINA-RG ensures the quality of item bank construction and assembly of tests, especially for large-scale assessments. Their greater precision also reduces the number of necessary test items to accurately assess a test-taker’s domain knowledge, thereby enabling more subdomains to be assessed. The RT-GDINA-RG results regarding time can also inform designers of timed tests regarding the time needed for different solution approaches to a test question. For example, for a timed test, items have frequent RG might because test-takers perceive that they lack sufficient time to attempt a solution. Thus, such information can provide the users of test scores to set a suitable time (e.g., increasing the response time) for completing the test. In addition, greater accuracy in the estimation of test scores increases users’ confidence in the results and their subsequent inferences.

When using RT-GDINA-RG to estimate more precise person and item parameters during RG, Q-matrix is an essential component in CDM contexts. An identifiable Q-matrix ensures the consistency of a CDM estimation. In this study, the simulation studies used an identifiable Q-matrix (Xu and Zhang, 2016; Xu, 2017), and the real data analysis adopted a partially identifiable Q-matrix (Gu and Xu, 2020). To enable consistent CDM estimation, checking the identifiability of the Q-matrix in advance is crucial. Besides, for ease of use, a tutorial to introduce the RT-GDINA-RG in JAGS can be developed in future work (cf. Curtis, 2010; Zhan et al., 2019).

## Data Availability Statement

The raw data supporting the conclusions of this article will be made available by the authors, without undue reservation.

## Author Contributions

All authors contributed to the article and approved the submitted version.

## Conflict of Interest

The authors declare that the research was conducted in the absence of any commercial or financial relationships that could be construed as a potential conflict of interest.
